# Indoor and outdoor residual spraying of a novel formulation of deltamethrin K-Othrine^®^ (Polyzone) for the control of simian malaria in Sabah, Malaysia

**DOI:** 10.1371/journal.pone.0230860

**Published:** 2020-05-15

**Authors:** A. Rohani, H. Ahmad Fakhriy, I. Suzilah, M. N. Zurainee, W. M. A. Wan Najdah, M. Mohd Ariffin, N. Mohamad Shakirudin, M. S. Mohd Afiq, J. Jenarun, Y. Tanrang, H. L. Lee

**Affiliations:** 1 Medical Entomology Unit & WHO Collaborating Centre for Vectors, Institute for Medical Research, Kuala Lumpur, Malaysia; 2 Parasitology Department, Faculty of Medicine, University Malaya, Kuala Lumpur, Malaysia; 3 School of Quantitative Sciences, Universiti Utara Malaysia, Sintok Kedah, Malaysia; 4 Sabah Department of Health, Kementerian Kesihatan Malaysia, Kota Kinabalu, Sabah, Malaysia; Kyung Hee University, REPUBLIC OF KOREA

## Abstract

Since 2000, human malaria cases in Malaysia were rapidly reduced with the use of insecticides in Indoor Residual Spray (IRS) and Long-Lasting Insecticide Net (LLIN). Unfortunately, monkey malaria in humans has shown an increase especially in Sabah and Sarawak. The insecticide currently used in IRS is deltamethrin K-Othrine^®^ WG 250 wettable granule, targeting mosquitoes that rest and feed indoor. In Sabah, the primary vector for knowlesi malaria is *An*. *balabacensis* a species known to bite outdoor. This study evaluates an alternative method, the Outdoor Residual Spray (ORS) using a novel formulation of deltamethrin K-Othrine^®^ (PolyZone) to examine it suitability to control knowlesi malaria vector in Sabah, compared to the current method. The study was performed at seven villages in Sabah having similar type of houses (wood, bamboo and concrete). Houses were sprayed with deltamethrin K-Othrine^®^ (PolyZone) at two different dosages, 25 mg/m^2^ and 30 mg/m^2^ and deltamethrin K-Othrine^®^ WG 250 wettable granule at 25 mg/m^2^, sprayed indoor and outdoor. Residual activity on different walls was assessed using standard cone bioassay techniques. For larval surveillances, potential breeding sites were surveyed. Larvae were collected and identified, pre and post spraying. Adult survey was done using Human Landing Catch (HLC) performed outdoor and indoor. Detection of malaria parasite in adults was conducted via microscopy and molecular methods. Deltamethrin K-Othrine^®^ (PolyZone) showed higher efficacy when sprayed outdoor. The efficacy was found varied when sprayed on different types of wall surfaces. Deltamethrin K-Othrine^®^ (PolyZone) at 25 mg/m^2^ was the most effective with regards to ability to high mortality and effective knock down (KD). The vector population was reduced significantly post-spraying and reduction in breeding sites as well. The number of simian malaria infected vector, human and simian malaria transmission were also greatly reduced.

## Introduction

Malaria is still one of the tropical diseases that post a great challenge as far as global health is concerned. In 2017, about 219 million cases reported and over 435 000 deaths globally [1]. In Malaysia malaria has achieved a 95% reduction in the number of reported cases for the past two decades, and Malaysia is categorized as in its pre-elimination phase by the World Health Organization (WHO) [1]. Although the incidence of human malaria is decreasing the incidence of knowlesi malaria in Malaysia appears to be on the rise [2]. Despite much government effort to reduce transmission of malaria, cases are still being recorded in rural areas of Sabah and Sarawak [3,4,5] with most of them are knowlesi malaria [6,7]. Now cases of knowlesi malaria have been reported throughout Malaysia and the cases are increasing year after year. Deaths due to knowlesi malaria were also been reported [3]. In Sabah, the primary vector for knowlesi malaria is *An*. *balabacensis* [8]. They are found abundant in rural areas inhabiting the forest and shown to rest and feed outdoor typically after dusk [8,9]. *An*. *balabacensis* is also an early biter and bites throughout the night, which makes its control using current control strategy challenging.

The current malaria vector control measures like indoor residual spraying (IRS) and long-lasting insecticide treated net (LLIN) [10] targeting only *Anopheles* that come in contact in the house and therefore miss out on the larger populations of mosquito that rest and feed outside the house. In addition areas where malaria cases still persists include aboriginal areas, tribal villages, and areas where communities working in agricultural/land development; these lands are located in rural areas where they are quite challenging to reach as they are without proper road, and to make matter worse house owners usually left for work very early in the morning and therefore access to the house has become another problem faced when performing IRS; moreover IRS required to be applied six monthly apart for maximum effect.

A study conducted in Sabah reported that the number of *An*. *balabacensis* that come into houses was much lower compared to those present outside [8,11]. It is important to consider such characteristic before performing vector control activities as it could cause the vector control used to be less suitable for the mosquito in the affected area like Sabah. Recently WHO has acknowledged that outdoor malaria transmission can persist despite comprehensive, population-wide coverage of IRS and LLINs with active ingredients to which local vector populations are fully susceptible [12,13]. Outdoor residual malaria transmission has been consistently reported in many areas where these interventions are in place [14,15]. The widely use of LLINs and IRS has also shown to cause an increase in insecticide resistance in the malaria vector mosquitoes both chemically [14,16] and behaviourally [17,18,19]; thus reducing the efficacy of these tools and increasing the outdoor malaria transmission.

Regardless of whether the mosquito behaviour represent resilience or resistance, elimination of malaria transmission from many endemic settings will require new or improved anti-vector measures that target mosquitoes when they feed outdoors upon humans or livestock, or at source in the aquatic habitats where their immature stages develop in [16,18]. There is a critical need to develop new vector control methods to address malaria parasite transmission that occurs outdoor. The recent development of a new formulation of pyrethroid known as deltamethrin K-Othrine^®^ (PolyZone) has provided a new tool for outdoor residual application. This new formulation is characterised by its anti-rain property, which makes it a suitable insecticide candidate for outdoor application. Study by Rohani *et*. *al*.,[20] demonstrated that different wall surface presented dissimilar results with regards to effectiveness of the insecticide sprayed in causing death to the mosquito [20]. It is therefore vital to also determine the suitable concentration of this new formulation based on wall type where spraying is to be conducted.

This study aims to evaluate the effectiveness of new formulation deltamethrin K-Othrine^®^ (PolyZone) as compared to currently used vector control which applied deltamethrin K-Othrine^®^ WG 250 wettable granule on different wall surfaces sprayed indoor and outdoor.

## Materials and methods

### Study area

**Fig 1:**
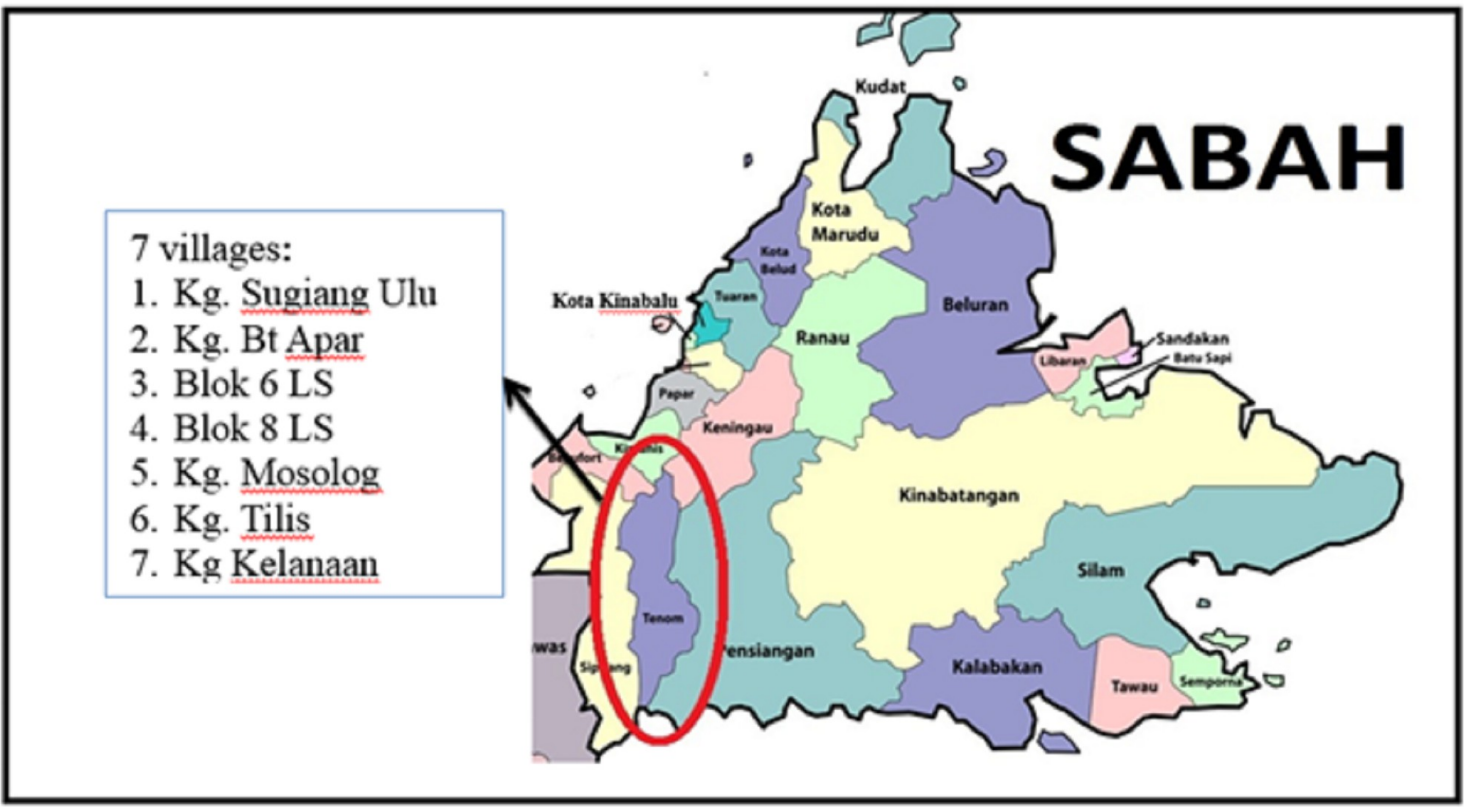
The location of the seven villages in Tenom, Sabah where the study was conducted.

The study was conducted in Tenom District (Fig 1) in the interior of Sabah. It is located 176 kilometres south of the state capital, Kota Kinabalu. Field trial was conducted in seven villages: Kg. Ulu Sugiang (N4° 55.035' E115° 53.166'), Kg. Bt Apar (N5° 12.265' E115° 57.098'), Blok 6 LS (N5° 12.237' E116° 01.152'), Blok 8 LS (N5° 12.986' E116° 00.983'), Kg. Mosolog (N5° 15.210' E116° 00.750'), Kg. Tilis (N4° 46.496' E115° 54.067') and Kg Kelanaan (N5° 11.934' E116° 02.428'). Villages were selected for the study based on human blood survey conducted in those villages that revealed high rate of *P*. *knowlesi* for several consecutive years, their similarity geographically and demographically.

In general, the villages are carved out of secondary forest situated on hilly terrain. Most of these villages are in remote areas and accessible only by four-wheel vehicle, while others can only be reached by foot. Most of the houses are surrounded by small farming areas in the rubber or oil palm plantations or near forest fringes. The houses in the villages consisted of individual, link and long houses and were built using various materials including wood, bamboo and concrete.

### Insecticides used and spraying activity

Two types of insecticide were used for the study: the new formulation deltamethrin K-Othrine^®^ (PolyZone) (a.i. 62.5 g/L deltamethrin) and currently used vector control insecticide, deltamethrin K-Othrine^®^ WG 250 wettable granule. For the new formulation deltamethrin K-Othrine^®^ (PolyZone), two dosages were tested: 25 mg/m^2^ and 30 mg/m^2^. This product has been approved for use by WHO [21]. For deltamethrine K-Othrine^®^ WG 250 wettable granule, only 25 mg/m^2^ was used which is the current dosage employed for malaria vector control. Spraying was conducted outdoor (ORS) on all surfaces outside the house (including area that is shaded and unshaded) and indoor (IRS) on all surfaces in the house that are potentially use by mosquito as resting place. The selection of household for each formulation and concentration was done based on distance of the villages. All houses from three nearby villages were sprayed with same formulation having the same concentration. The spraying were performed as follows: All houses from three nearby villages namely Kampung Kelanaan, Blok 6 LS and Blok 8 LS were sprayed with 25 mg/m^2^ deltamethrin K-Othrine^®^ (PolyZone); while houses from another three nearby villages, Kampung Ulu Sugiang, Kampung Mosolog and Kampung Batu Apar were sprayed with 30 mg/m^2^ deltamethrin K-Othrine^®^ (PolyZone) and finally 25 mg/m^2^ deltamethrine K-Othrine^®^ WG 250 wettable granule was used to spray on all houses from the final village, Kampung Tilis which were used as control houses. The study was conducted within the 18 months period (73 weeks) which consisted of two cycles of spraying. First spraying was conducted in week 1 and second spraying in week 37 for all study villages. Spraying was performed by Ministry of Health trained and experienced staff using Hudsons Compressor Sprayer according to WHO guidelines and manufacturer instruction.

### Experimental design

**Fig 2:**
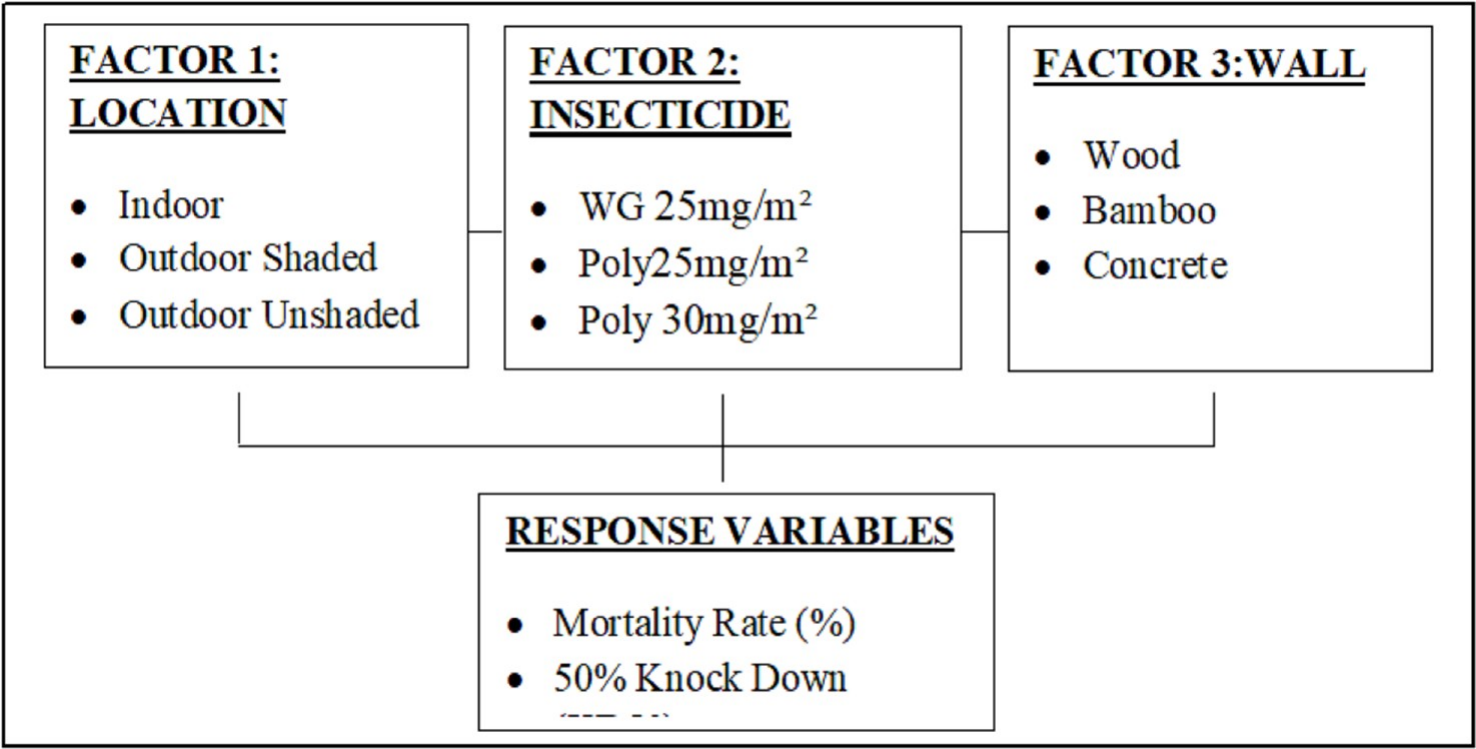
Experimental framework outline.

Factorial design was used involving three factors which are location (indoor, outdoor shaded area and outdoor unshaded area), type of insecticide (deltamethrin K-Othrine^®^ WG 250 wettable granule at 25mg/m^2^, deltamethrin K-Othrine^®^ (PolyZone) at 25mg/m^2^ and at 30mg/m^2^) and type of wall (wood, bamboo and concrete). The response variables are mortality rate and 50% knock down (KD50). The outline of the experimental framework is shown in (Fig 2).

Residual activity on different walls was assessed by using standard World Health Organization (WHO) cone bioassay techniques [22]. In this study, *An*. *balabacensis* laboratory strain was used for the purpose of adult bioassay test since *An*. *balabacensis* is the primary vector for both human and zoonotic malaria in the study area. For each study village, only nine (three/house type) houses were randomly selected as “index houses” to perform the residual bioassay test in order to determine the residual efficacy of the insecticide sprayed.

Residual activity was monitored every 6 weeks for 18 months (73 weeks) post spraying. All tests were conducted at the index houses. Briefly, cones were affixed onto the sprayed wall. A bioassay of five replicates for each dose and wall was conducted. Twenty (20) sugar-fed 2–5 days old laboratory-bred *An*. *balabacensis* female mosquitoes were released into each cone through the aperture which was then clogged with cotton bud. Mosquitoes were exposed continuously for 30 minutes to the sprayed wall. During the test, cones were covered with black cloth to reduce disturbance caused by light in order to calm the mosquitoes down. Knock down was recorded every three minutes until a period of 30 minutes. KD50 values were then calculated using probit analysis software. After exposure was completed, the live mosquitoes were transferred to paper cups where 10% sugar solution and Vitamin B complex were supplied to the mosquitoes. They were then kept for 24 hours where the mortality rate was recorded and analysed using ANOVA.

Bionomic of mosquito was conducted through the larval survey, adult survey and environmental survey. For larval surveillances, all potential breeding sites were surveyed. Larvae were collected and identified, pre and post spraying. Alive mosquito larvae were collected in a bottle. In the laboratory *Anopheles* mosquitoes were identified using the keys of Reid [23] and Sallum [24]. Specimens morphologically identified as *An*. *balabacensis* were further confirmed by PCR and sequencing analysis of ITS2 [25]. Breeding sites and their distance to the nearest house were recorded using GPS. Environmental parameters were recorded by collecting the data of rainfall, temperature and humidity by installing localize weather station at study villages.

For adult survey, adult mosquitoes in all seven villages were also caught every 6 weeks for the period of 18 months outdoor and indoor using Human Landing Catching (HLC) technique. The collection was carried out from 6:00 PM to 6:00 AM (12 hours duration) for three straight days for every village. Mosquito collectors worked in pairs and in shifts of 6 hours. Collected mosquitoes were recorded hourly. The location for outdoor HLC was determined based on space availability near to breeding site found during larvae surveillance and the sampling sites were then marked using GPS, while indoor HLC were done in the index houses.

All mosquito captured were then identified to species level. They were then dissected to detect presence of malaria parasite via microscopy and molecular methods. Briefly, mosquitoes were dissected to remove the salivary gland and examined microscopically for the presence of sporozoite; while the mid-gut and the ovary were also examined for the presence of oocyst. The sporozoite and oocyst obtained were then transferred into 1.5ml microcentrifuge tubes containing 95% alcohol and labelled accordingly. The tubes were later transported to the Institute of Medical Research (IMR) laboratory for molecular identification of malaria parasite species by using semi-nested and nested PCR [26,27] techniques. The entomological and epidemiological endpoints were residual activity of the formulation, changes in vector population and breeding sites, and impact on simian malaria transmission.

### Ethics

Informed consent was obtained from all collectors performing Human Landing Catching (HLC). Community consent had been obtained beforehand from all selected villages. This study was approved by Medical Research & Ethics Committee, Ministry of Health, Malaysia (NMRR-16-318-29507).

## Results and discussions

**Fig 3:**
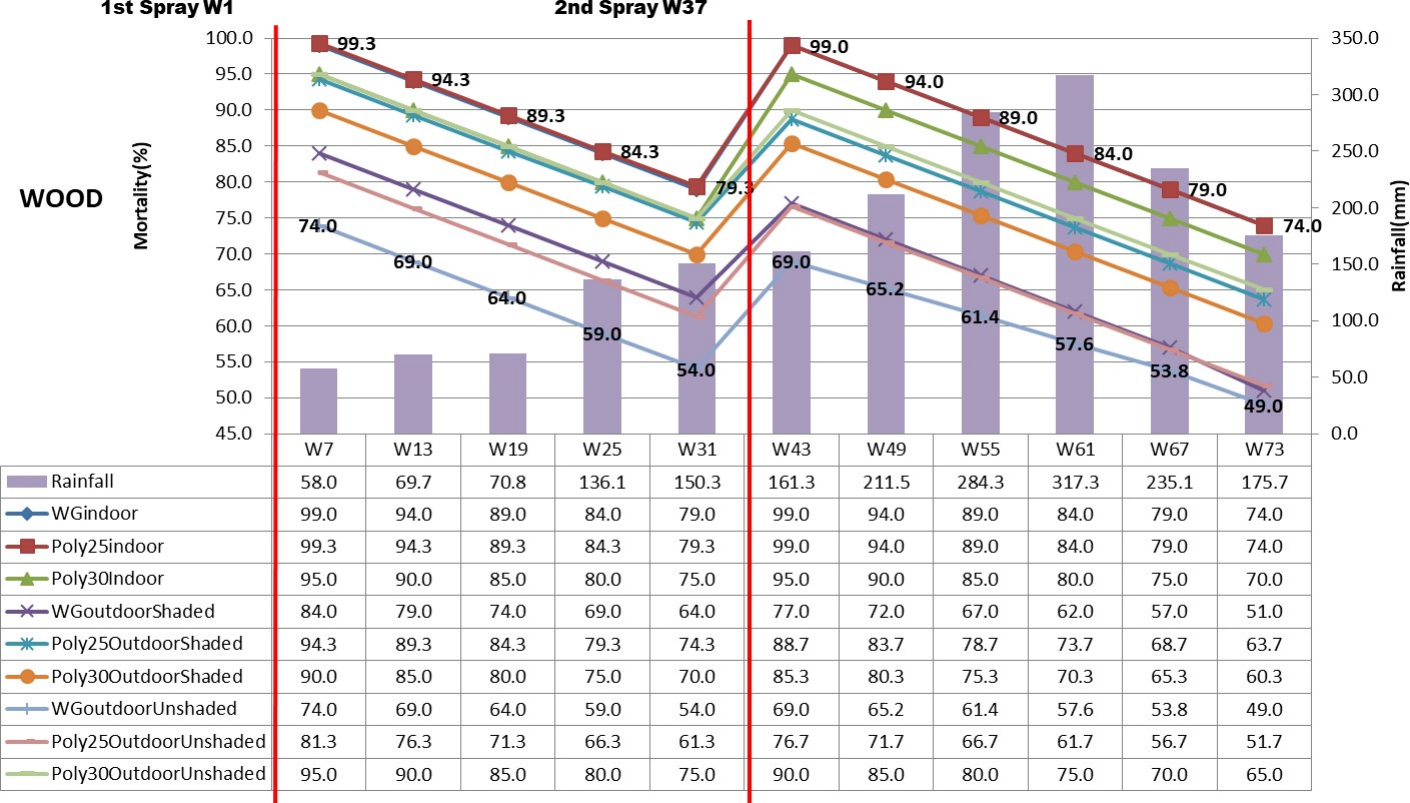
Mortality rate (%) based on location, insecticide, wood wall and rainfall.

**Fig 4:**
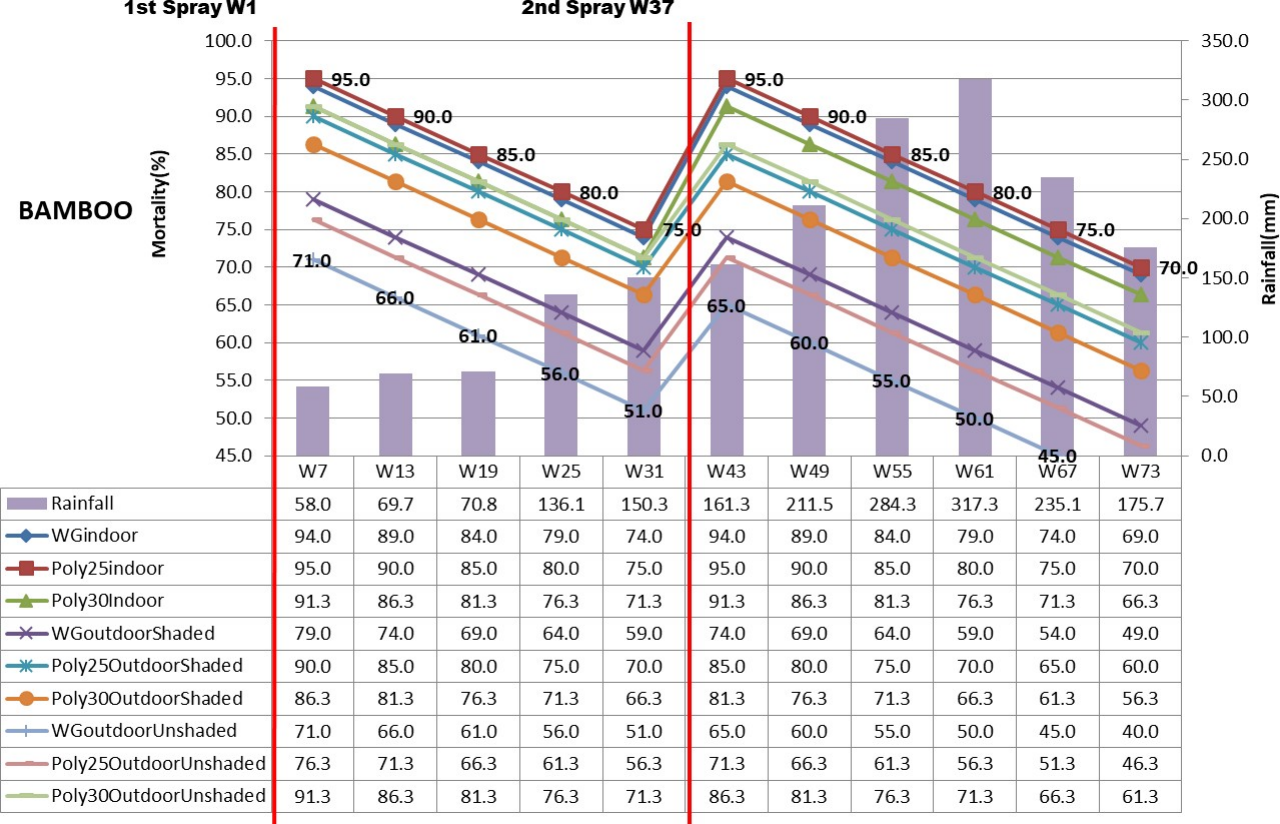
Mortality rate (%) based on location, insecticide, bamboo wall and rainfall.

**Fig 5:**
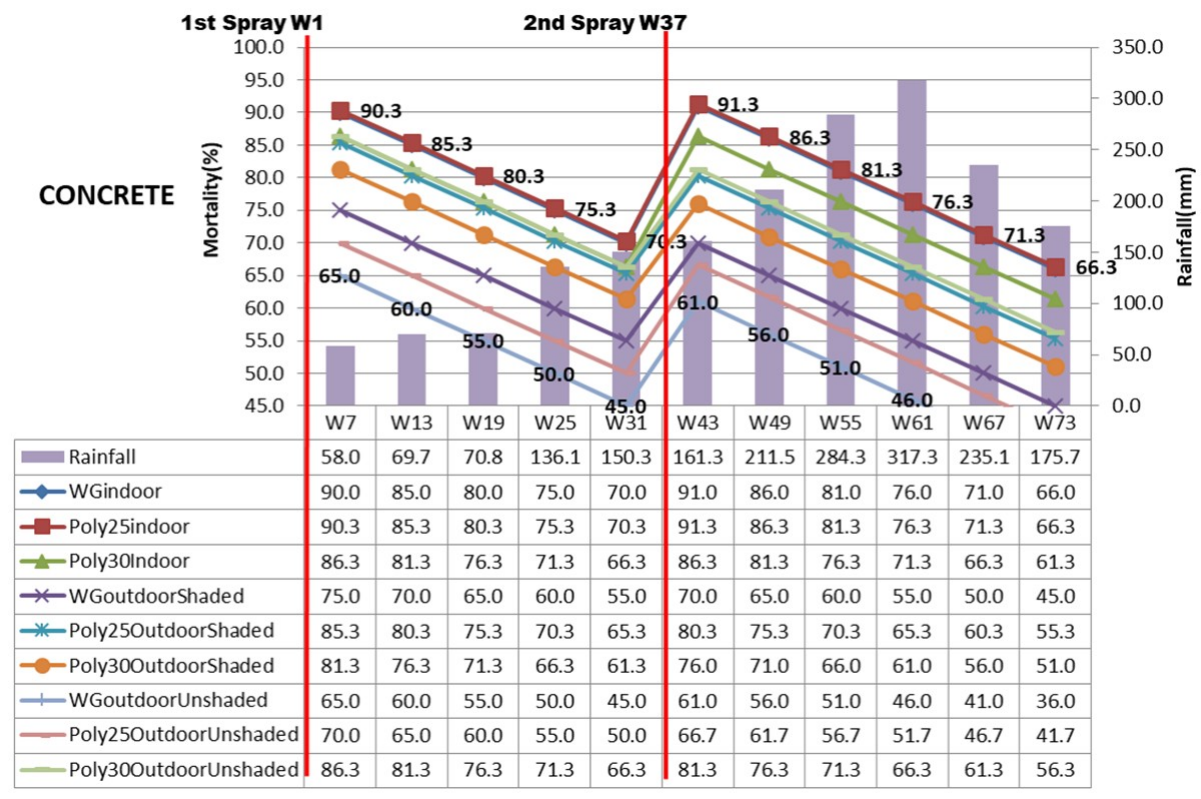
Mortality rate (%) based on location, insecticide, concrete wall and rainfall.

The mortality rate of mosquito shown in Figs 3–5 displayed the mosquito mortality rate (%) based on spraying location (indoor, outdoor unshaded and outdoor shaded areas), type of insecticide (deltamethrin K-Othrine^®^ (PolyZone) and deltamethrin K-Othrine^®^ WG 250 wettable granule), type of wall (wood, bamboo and concrete) and rainfall at 6 weekly intervals. The first spraying was conducted in week 1 and second spraying was implemented in week 37. Deltamethrin K-Othrine^®^ (PolyZone) at 25mg/m^2^ sprayed indoor caused highest mortality rate, while deltamethrin K-Othrine^®^ WG 250 wettable granule at 25mg/m^2^ sprayed on outdoor unshaded area showed the lowest mortality rate. However it was noticeable that deltamethrin K-Othrine^®^ WG 250 wettable granule at 25mg/m^2^ sprayed indoor caused similar mortality rate as deltamethrin K-Othrine^®^ PolyZone at 25mg/m^2^ sprayed indoor while deltamethrin K-Othrine^®^ (PolyZone) at 30mg/m^2^ sprayed indoor caused lower mortality. The mortality rates deteriorated about 5% every 6 weeks. In the first spraying duration (week 1–36), when comparing deltamethrin K-Othrine^®^ WG 250 wettable granule at 25mg/m^2^ sprayed indoor and deltamethrin K-Othrine^®^ WG 250 wettable granule at 25mg/m^2^ sprayed outdoor shaded and unshaded area; the mortality rate deteriorated about 15% and 25% every 6 weeks respectively. However the deltamethrin K-Othrine^®^ (PolyZone) at 25mg/m^2^ sprayed indoor and outdoor shaded and unshaded areas, caused mortality rate to deteriorate only about 5% and 18% respectively. Surprisingly deltamethrin K-Othrine^®^ (PolyZone) at 30mg/m^2^ sprayed indoor caused similar mortality rate with deltamethrin K-Othrine^®^ (PolyZone) at 30mg/m^2^ sprayed outdoor unshaded area but about 5% lower as compared to deltamethrin K-Othrine^®^ (PolyZone) at 30mg/m^2^ sprayed outdoor shaded. In the second spraying duration (week 37- week 73) the deteriorations of mortality rate were faster about 22 to 30% for deltamethrin K-Othrine^®^ WG 250 wettable granule at 25mg/m^2^; 10 to 22% for deltamethrin K-Othrine^®^ (PolyZone) at 25mg/m^2^; and 5% between deltamethrin K-Othrine^®^ (PolyZone) at 30mg/ m^2^ when sprayed indoor and outdoor unshaded area but lower about 10% as compared to deltamethrin K-Othrine^®^ (PolyZone) at 30mg/m^2^ sprayed outdoor shaded area. The mortality rate deterioration of the same insecticide sprayed outdoor (shaded and unshaded areas) in the first and second spray durations was likely due to heavy rainfall recorded in the second spraying duration (i.e. mortality rate has significant negative correlation with rainfall but not temperature and humidity). The mortality rate also differed based on different type of wall surface whereby wood showed the highest mortality rate, followed by bamboo and then concrete. This finding clearly indicates that the residual efficacy of deltamethrin varied when sprayed on different type of surfaces. Since spraying was done by trained and experienced staff of Ministry of Health Malaysia and conducted according to WHO guidelines and manufacturer instruction, it was strongly believed that quality of spraying or quality of insecticide required for all houses during 1^st^ spraying and 2^nd^ spraying was satisfactorily achieved and therefore was not considered as factor that could have influence on the result obtained.

The residual effect of deltamethrin, like any other insecticides, depends on the nature of treated surface and the type of formulation used [28,29,30]. Synthetic pyrethroids are found commonly very stable on wood and bamboo, and thus providing good residual efficacy and longer duration of residual effect while rough and porous surface (highly alkaline surface) tends to absorb the insecticide resulted in shorter duration of residual effect [20,30,31,32]. Concrete surface is porous and may degrade the molecule of the insecticides faster [33].

There are several reports on the efficacy of deltamethrin on different surfaces against different species of malaria vectors worldwide [20,28,34–38]. Results showed that the efficacy of deltamethrin mainly depends on spraying location, concentration of insecticides used, formulation, the surface, humidity, temperature and method of evaluation. The persistency of insecticide, as revealed by mortality, also depended on the sustained insecticide [39].

**Fig 6:**
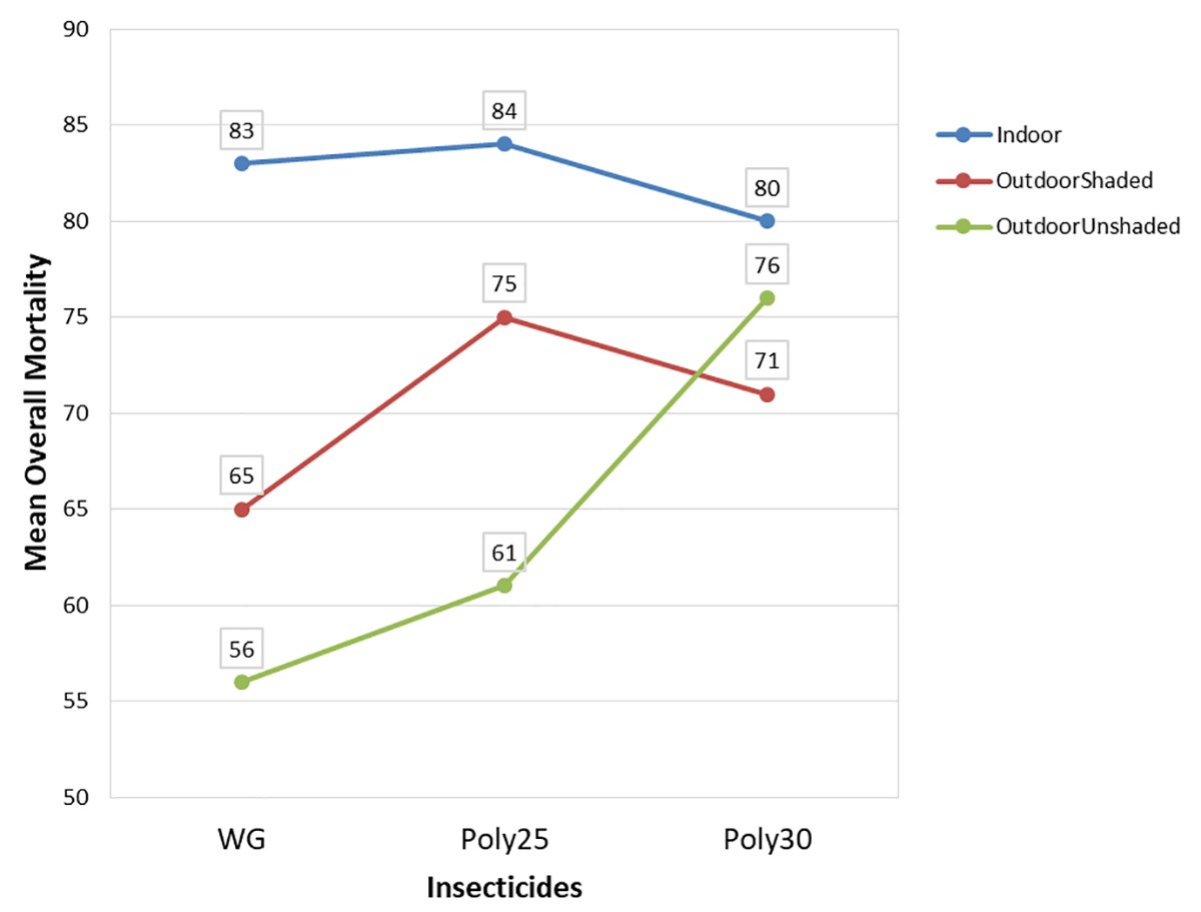
Average overall mortality based on insecticide and location.

Since the mortality rates deteriorate according to weeks, ANOVA was conducted separately for every 6 weeks and the summary shown in Table 1. Based on week 7 until week 73, there were significant interactions between spraying location and insecticide and also significant main effects (spraying location, insecticide and wall) across the weeks. Thus, overall mortality was calculated by pooling the mortality rate data from week 7 until week 73 by using median and ANOVA was performed which indicated similar results. Fig 6 highlighted the significant interaction between spraying location and insecticide where deltamethrin K-Othrine^®^ (PolyZone) at 30mg/m^2^ sprayed outdoor unshaded area performed better than deltamethrin K-Othrine^®^ (PolyZone) at 30mg/m^2^ sprayed outdoor shaded area. Shaded area was less affected by the rainfall and therefore insecticide concentration was maintained compared to outdoor unshaded. Deltamethrin K-Othrine^®^ PolyZone at 30mg/m^2^ could cause excito-repellent effect to occur and thus repel mosquito from resting on treated wall resulted in lower mortality. Pyrethroids have well documented excito-repellent actions [40] which are dose dependent [41]. Studies also showed that mosquitoes possess a high degree of irritability or repellence and therefore are able to avoid further contact and escaped unharmed [16,42].

**Table 1:** Summary of ANOVA (p-values) based on weeks and overall mortality.

Factors	WEEKS											Overall Mortality
	W7	W13	W19	W25	W31	W43	W49	W55	W61	W67	W73	
Location/Insecticide/Wall/ Location*Insecticide	0.000**	0.000**	0.000**	0.000**	0.000**	0.000**	0.000**	0.000**	0.000**	0.000**	0.000**	0.000**
Location*Wall	0.798	0.786	0.718	0.783	0.766	0.667	0.682	0.636	0.543	0.426	0.310	0.889
Insecticide*Wall	0.731	0.723	0.730	0.746	0.715	0.638	0.494	0.353	0.238	0.157	0.186	0.640
Location*Insecticide *Wall	0.952	0.912	0.925	0.912	0.928	0.765	0.880	0.930	0.940	0.921	0.862	0.987

0.000** indicated the p-value is less than 0.000001 which is highly significant at 1%

Next individual ANOVA and Bonferroni post hoc test based on each location was conducted in determining deeper understanding about the factors. Table 2 presents the summary of ANOVA and Boferroni post hoc test results according to each spraying location. Insecticides and walls were significant. For indoor spraying, based on Boferroni post hoc test, the mortality rate cause by deltamethrin K-Othrine^®^ WG 250 wettable granule at 25mg/m^2^ and deltamethrin K-Othrine^®^ (PolyZone) at 25mg/m^2^ was similar but causing the significant difference in the insecticide was deltamethrin K-Othrine^®^ (PolyZone) at 30mg/m^2^ where it has lower mortality rate as indicated in Fig 6. As for spraying done on outdoor shaded and unshaded areas, the ANOVA and Boferroni post hoc test shows all the three insecticides have different mortality rate with deltamethrin K-Othrine^®^ (PolyZone) at 25mg/m^2^ performed the best when sprayed outdoor shaded area while deltamethrin K-Othrine^®^ (PolyZone) at 30mg/m^2^ performed the best when sprayed outdoor unshaded area. Bonferroni post hoc test also revealed all the three walls either sprayed indoor or outdoor shaded or unshaded areas have different mortality rate where the insecticide last longer on wood, followed by bamboo and lastly concrete.

**Table 2:** Summary of ANOVA (p-values) for overall mortality rate according to each location.

Location	Factor	ANOVA (p-value)	Post Hoc Test (Bonferroni p-value)		
**Indoor**	Insecticide	0.000**	WG	Poly25	0.978
				Poy30	0.000**
			Poly25	WG	0.978
				Poly30	0.000**
			Poly30	WG	0.000**
				Poly25	0.000**
	Wall	0.000**	Wood	Bamboo	0.000**
				Concrete	0.000**
			Bamboo	Wood	0.000**
				Concrete	0.000**
			Concrete	Wood	0.000**
				Bamboo	0.000**
	Insecticide*Wall	0.888	-		
**Outdoor Shaded**	Insecticide	0.000**	WG	Poly25	0.000**
				Poy30	0.000**
			Poly25	WG	0.000**
				Poly30	0.000**
			Poly30	WG	0.000**
				Poly25	0.000**
	Wall	0.000**	Wood	Bamboo	0.000**
				Concrete	0.000**
			Bamboo	Wood	0.000**
				Concrete	0.000**
			Concrete	Wood	0.000**
				Bamboo	0.000**
	Insecticide*Wall	0.692	-		
**Outdoor Unshaded**	Insecticide	0.000**	WG	Poly25	0.000**
				Poy30	0.000**
			Poly25	WG	0.000**
				Poly30	0.000**
			Poly30	WG	0.000**
				Poly25	0.000**
	Wall	0.000**	Wood	Bamboo	0.000**
				Concrete	0.000**
			Bamboo	Wood	0.000**
				Concrete	0.000**
			Concrete	Wood	0.000**
				Bamboo	0.000**
	Insecticide*Wall	0.731	-		

0.000** indicated the p-value is less than 0.000001 which is highly significant at 1%

**Fig 7:**
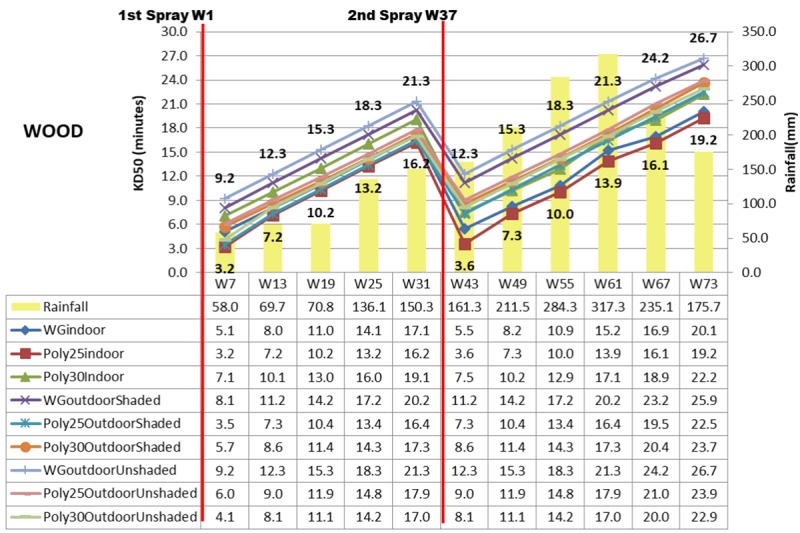
KD50 in Minutes based on location, insecticide, wood wall and rainfall.

**Fig 8:**
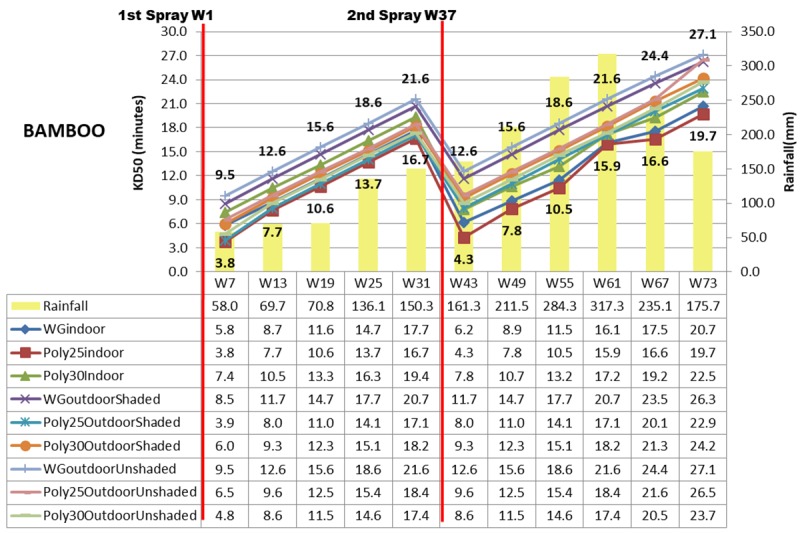
KD50 in Minutes based on location, insecticide, bamboo wall and rainfall.

**Fig 9:**
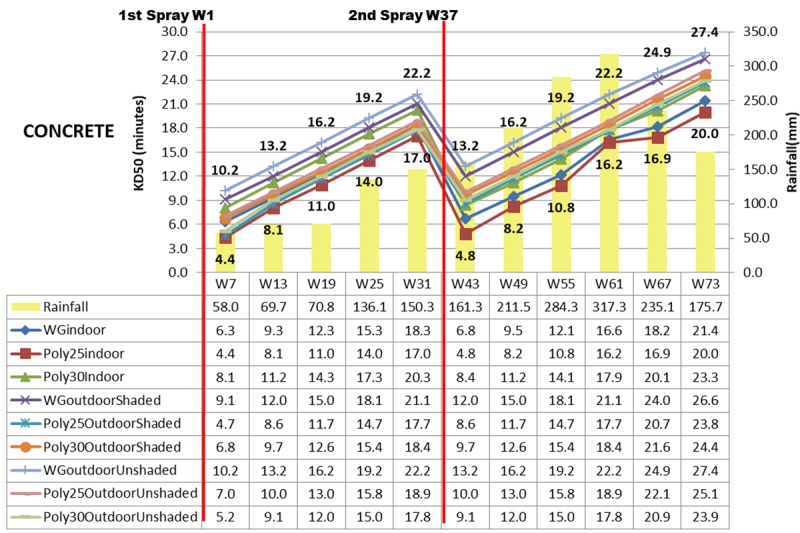
KD50 in Minutes Based on location, insecticide, concrete wall and rainfall.

Other than mortality rate, 50% knock down (KD50) in minutes was computed by using probit analysis as shown in Figs 7–9. Deltamethrin K-Othrine^®^ (PolyZone) at 25mg/m^2^ sprayed indoor has the fastest KD50 ranging from 3.2 minutes in week 7 to 19.2 minutes in week 73 while deltamethrin K-Othrine^®^ WG 250 wettable granule sprayed outdoor unshaded area has the slowest effect (from 9.2 minutes up to 26.7 minutes) as shown in Fig 7. All the three insecticides have significantly different KD50 when sprayed either indoor, outdoor shaded area or outdoor unshaded area and also based on different types of wall. Deltamethrin K-Othrine^®^ (PolyZone) at 25mg/m^2^ performed the best indoor and outdoor shaded area but deltamethrin K-Othrine^®^ (PolyZone) at 30mg/m^2^ was the best for outdoor unshaded area. Similar to mortality rate KD50 of insecticide sprayed outdoor (shaded and unshaded areas) was also significantly affected by heavy rainfall that occurred in the second spraying duration period (an increment of 3 to 5 minutes). As for the type of wall KD50 was fastest for insecticide sprayed on wood, followed by bamboo and lastly concrete with the time range of 0.5 to 3 minutes.

**Fig 10:**
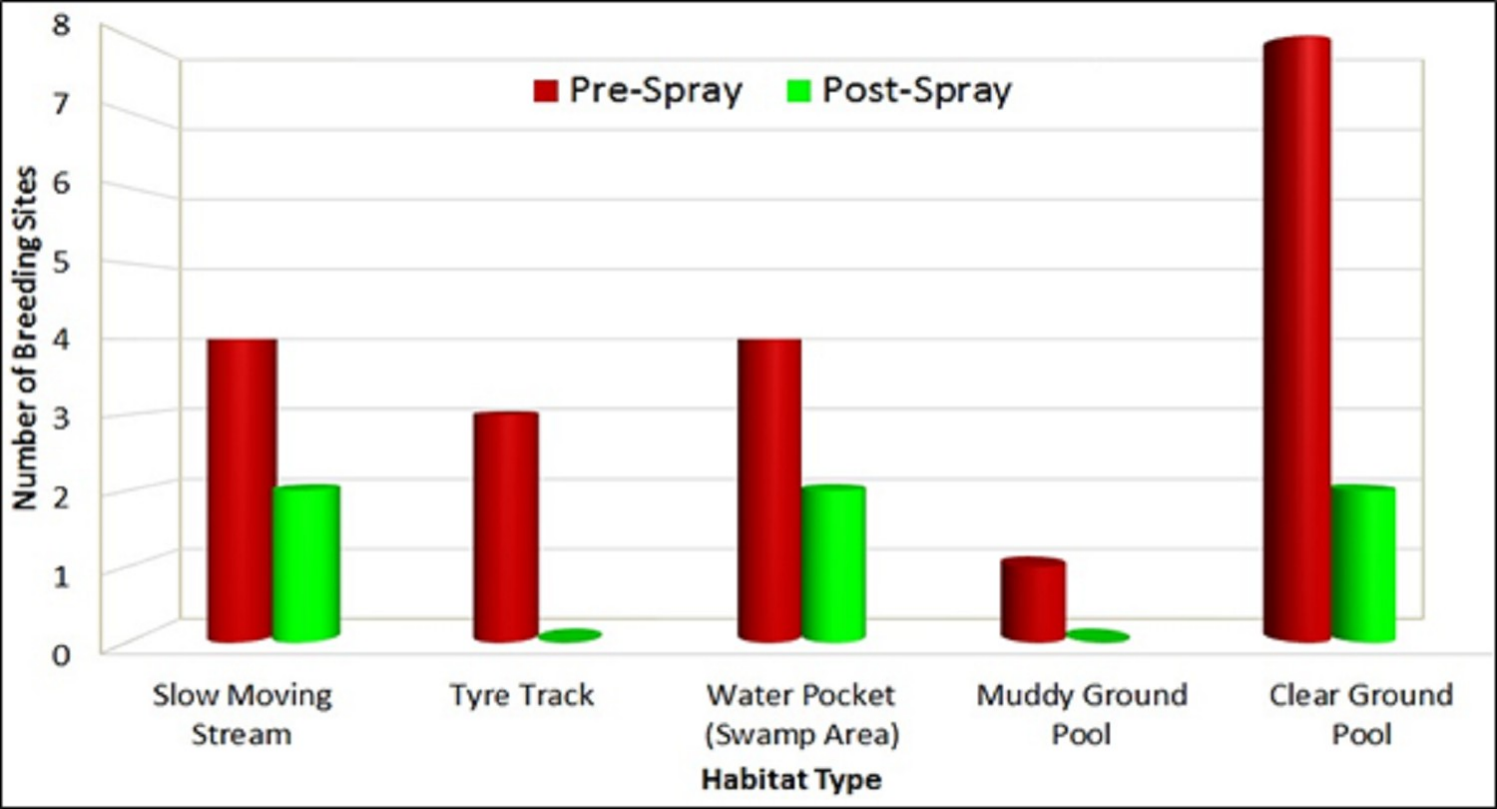
Distribution of breeding habitats of *An*. *balabacensis*.

Larvae surveillance conducted detected *An*. *balabacensis* breed in slow moving stream, tyre track, water pocket, muddy ground pool and clear ground pool (Fig 10). Similar study, conducted in Kudat and Penampang Sabah have also demonstrated similar breeding habitats for *An*. *balabacensis* [43,44]. Fig 10 also demonstrated the impact of insecticide spraying on the number of breeding sites where tremendous reduction in the number of breeding sites was observed, due to the reduced vector population.

**Fig 11:**
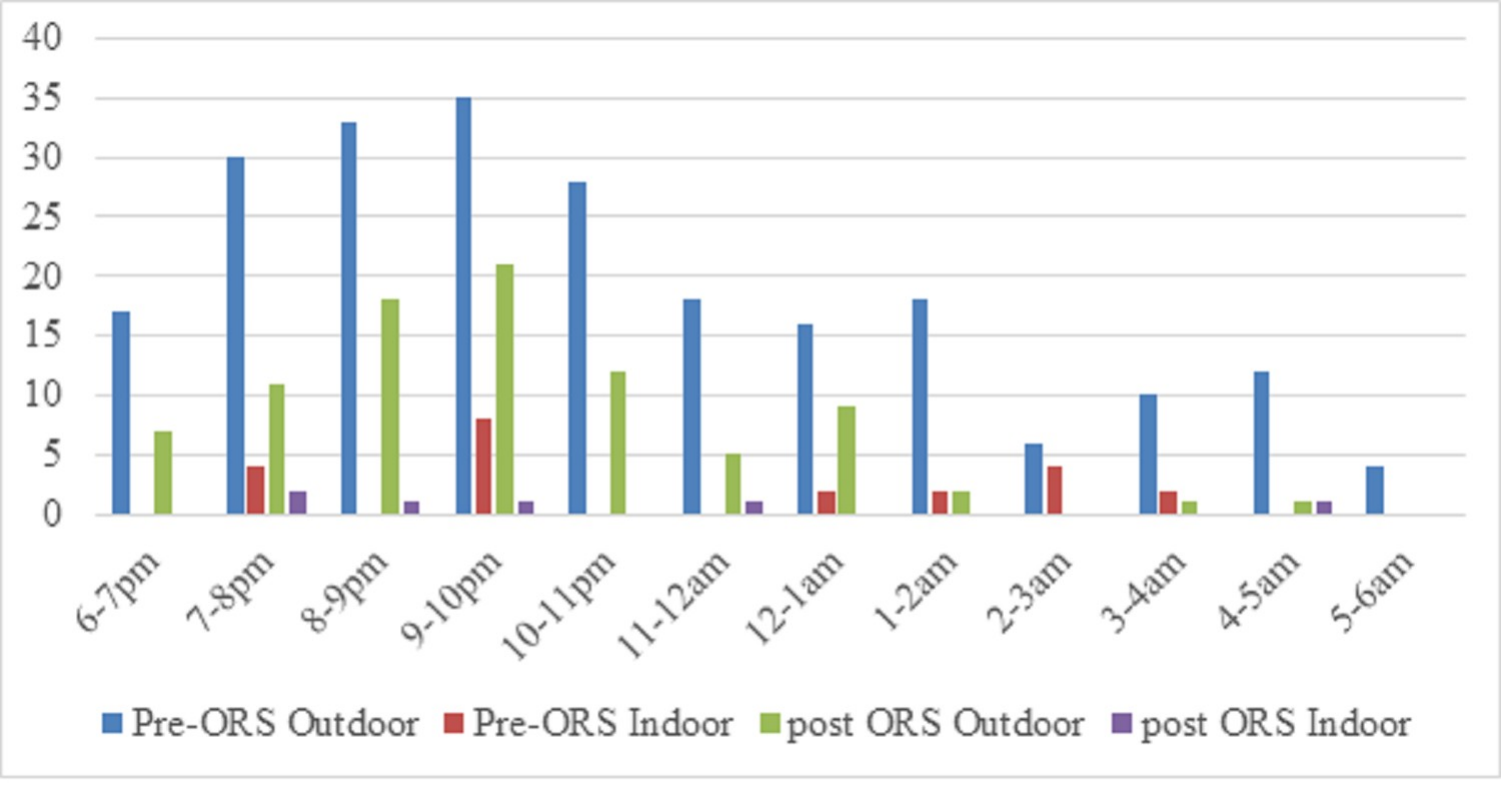
Outdoor and indoor biting activities of *An*. *balabacensis* before and after spraying.

Fig 11 demonstrated the detail of outdoor and indoor biting activities of *An*. *balabacensis* where *An*. *balabacensis* was found to be an early biter, biting as early as at 6.00 pm and shown to bite throughout the night with several peaks of biting activity. This present study also found that *An*. *balabacensis* showed preference towards biting outdoor than indoor, as 11.2 times more *An*. *balabacensis* were captured outdoor (n = 314) than indoor (n = 28) (Table 3A).

**Table 3:** Number of mosquitoes collected during the study.

3 (a) Number of mosquito captured	
Outdoor	Indoor
314	28
3 (b) Number of mosquito captured	
pre spraying	post spraying
249	93
3 (c) Number of mosquito captured	
pre spraying infected with malaria parasite	post spraying infected with malaria parasite
42	3

**Fig 12:**
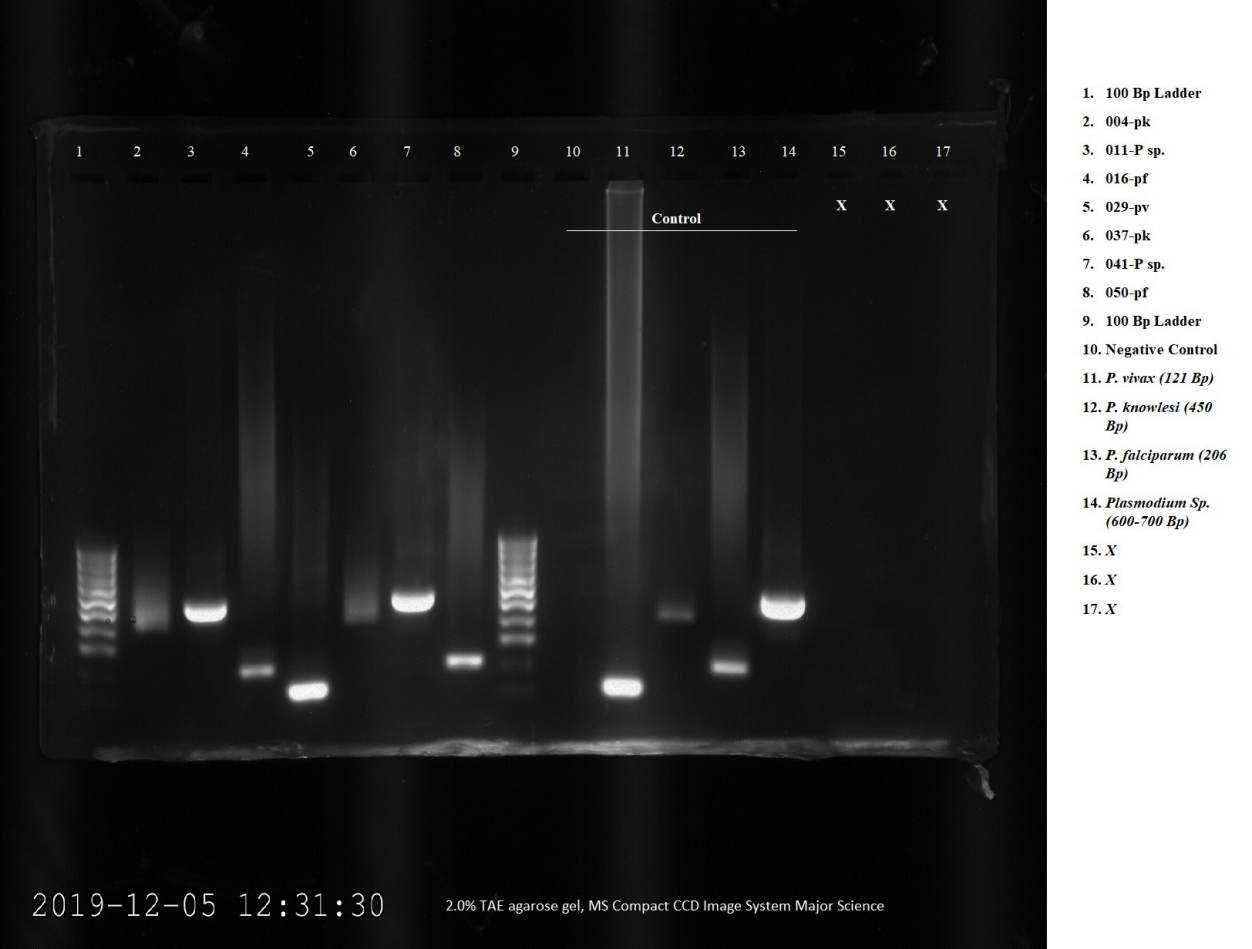
Malaria parasites species detected in *An*. *balabacensis* mosquito.

This study also examined the presence of malaria parasite in the mosquito captured. Out of 249 *An*. *balabacensis* captured before insecticide spraying 42 (16.87%) were found infected with malaria parasite. Only 93 *An*. *balabacensis* were captured after insecticide spraying and only 3 (3.22%) of them were found positive for malaria parasites (Table 3B and 3C). Clearly, residual activity during the study not only causes reduction in the number of mosquitoes vector collected, but had led to the low number of infected vector mosquitoes captured. Besides *P*. *knowlesi* other malaria parasites were also detected in the *An*. *balabacensis* mosquitoes. Molecular detection showed *An*. *balabacensis* was infected with *Plasmodium knowlesi*, *P*. *falciparum*, *P*. *vivax* and *Plasmodium* species (Fig 12).

**Fig 13:**
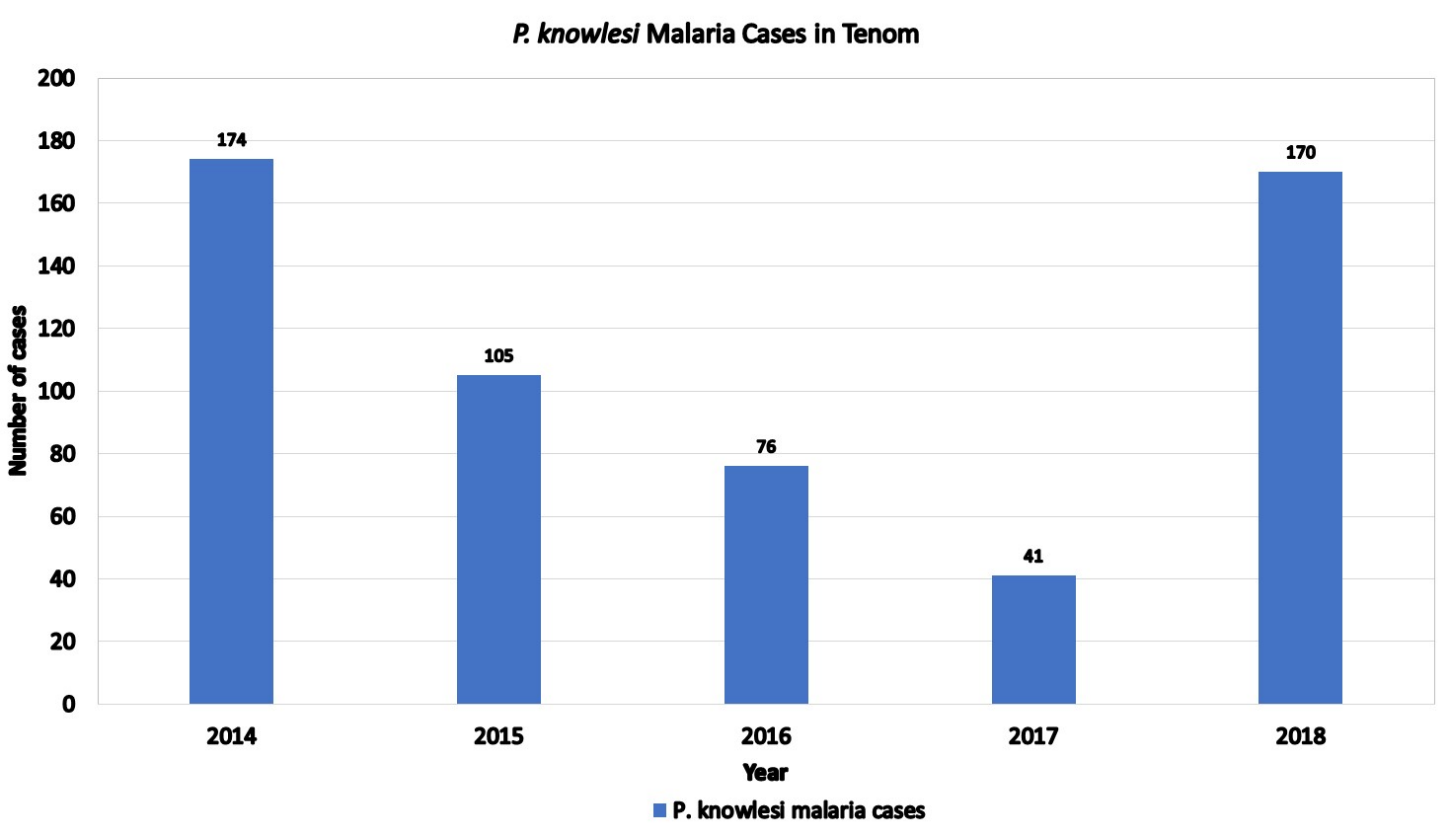
*P. knowlesi* malaria cases in Tenom, Sabah.

Fig 13 displays the yearly knowlesi malaria cases recorded in Tenom from 2014 to 2018. The malaria cases were shown to reduce significantly between year 2016 and 2017 which was during the study period. After the study was completed, the number of malaria cases shown to increase quiet drastically. Clearly this study has helped bringing down the number of malaria cases in Tenom and possibly an indication that ORS is likely effective in controlling the malaria vector in Tenom.

Data presented in this study demonstrated that deltamethrin K-Othrine^®^ PolyZone with enhanced formulation technology provides a longer residuality even when sprayed outdoor especially covered areas, such as roofed terraces where local populations meet to eat or for leisure. Efficacy levels were lower on unshaded outside walls, mainly due to heavy rainfall. Deltamethrin K-Othrine^®^ (PolyZone) extents the effective mosquito control both in indoor on difficult aggressive surfaces, as well as in outdoor conditions under the influence of precipitation. To the best of our knowledge, the study described here is the first of its kind in Asia and is generating a lot of interest, especially the impact on simian malaria transmission.

This study showed that *An*. *balabacensis* in Tenom, Sabah mainly remains and bites outside dwellings, similarly, current evidence indicates that previously incriminated mosquito vector of *P*. *knowlesi* in Malaysia bite and rest outdoors [8] where control methods such as LLINs and IRS will not be effective [25,45,46]. Outdoor transmission and biting immediately after dusk and early morning hours continue to pose a major prevention and control challenge. Based on our observation during the field trial, the knowlesi malaria is also to some extent related to the workers working in the forest fringe during dawn or very early in the morning tapping rubber. These people also live in the rubber plantations or in the forest fringe and their houses are just a base minimum with temporary walls which clearly does not offer them much of protection against mosquito.

New vector control tools therefore should be designed to target outdoor and early biting and feeding vector mosquitoes. Moreover, they should be accessible and acceptable for the population at risk. By combining both IRS and ORS vector control interventions, it is likely that vector mosquito population will be greatly reduced even after the efficacy period of the interventions in certain conditions, leading to long-term mosquito control and reduction of infected mosquitoes and cases. Although implementation of such new approaches might not be cost-effective, nevertheless they will be crucial if malaria elimination is the final goal.

## Conclusions

This study concludes that deltamethrin K-Othrine^®^ PolyZone at 25 mg/m^2^ was the most effective, giving rise to high adult mosquito mortality and effective knock down in indoor and outdoor shaded area. Deltamethrin K-Othrine^®^ PolyZone at 30 mg/m^2^ was the most effective, giving rise to high adult mosquito mortality and effective knock down for outdoor unshaded area. Residual efficacy of even the same dose of deltamethrin K-Othrine^®^ PolyZone varied when sprayed on different types of wall where the maximum residual efficacy was on wood, followed by bamboo, and lastly by concrete. The vector population and breeding sites were reduced significantly, and the malaria infected mosquito population and malaria cases were also reduced concomitantly. Therefore, indoor and outdoor residual spraying with the new formulation of deltamethrin is potentially an effective mean of controlling human and simian malaria vector in Sabah.

In the case where outdoor residual spraying is to be implemented for simian malaria control program in Sabah, this study recommended the use of deltamethrin K-Othrine^®^ PolyZone at 25mg/m^2^ be sprayed on outdoor shaded area while deltamethrin K-Othrine^®^ PolyZone at 30mg/m^2^ be sprayed on outdoor unshaded area.

This study faced several limitations that have caused some delay during visits made to almost all study villages. It involved condition of the road to these villages which was always in a very bad shape making it very difficult and dangerous to pass through even by 4-wheel vehicle. Similarly access to index houses to perform insecticide efficacy assessment is not always possible due to house owner not available at all time. More than one visit sometime is required just to have assessment done at single index house.

## Supporting information

S1 Fig (TIFF)

S2 Fig (TIF)

S1 Raw Images (PDF)

## References

[1] World Health Organization (WHO). Regional Office for the Western Pacific. (‎2018) *World Malaria Reports* Available from https://apps.who.int/iris/bitstream/handle/10665/275867/9789241565653-eng.pdf?ua=1.

[2] Minister of Health Malaysia. *Zoonotic malaria and the prevention program in Malaysia* [press release] (2018 November 7). Available from: http://www.moh.gov.my/index.php/database_stores/attach_download/337/1087

[3] Singh B, Sun LK, Matuso A, Radhakrishnan A, Shamsul SS, Cox-Singh J, et al A large focus of naturally acquired *Plasmodium knowlesi* infections in human beings. *The Lancet*. 2004;363(9414): 1017–1024. 10.1016/S0140-6736(04)15836-4 15051281

[4] Singh B, Daneshvar C. Human infections and detection of *Plasmodium knowlesi*. *Clinical microbiology reviews*.2013; 26(2): 165–184. 10.1128/CMR.00079-12 23554413 PMC3623376

[5] Rajahram GS, Barber BE, William T, Menon J, Anstey NM, and Yeo TW. Deaths due to *Plasmodium knowlesi* malaria in Sabah, Malaysia: association with reporting as *Plasmodium malariae* and delayed parenteral artesunate. *Malaria Journal*. 2012;11(1): 284 10.1186/1475-2875-11-284 22905799 PMC3472242

[6] Lau YL, Tan LH, Chin LC, Fong MY, Noraishah MAA, Rohela M. *Plasmodium knowlesi* reinfection in human. *Emerging infectious diseases*. 2011; 17(7): 1314 10.3201/eid1707.101295 21762601 PMC3381378

[7] Barber BE, William T, Dhararaj P, Anderios F, Grigg MJ, Yeo TW, et al Epidemiology of *Plasmodium knowlesi* malaria in north-east Sabah, Malaysia: family clusters and wide age distribution. *Malaria Journal*. 2012; 11(1): 401. 23216947 10.1186/1475-2875-11-401 PMC3528466

[8] Wong ML, Chua TH, Leong CS, Khaw LT, Fornace K, Wan-Sulaiman WY, et al Seasonal and Spatial Dynamics of the Primary Vector of Plasmodium *knowlesi* within a Major Transmission Focus in Sabah, Malaysia. *PLoS Neglected Tropical Diseases*.2015; 9(10): 1–15. 10.1371/journal.pntd.0004135 26448052 PMC4598189

[9] Sinka ME, Bangs MJ, Manguin S, Chareonviriyaphap T, Patil AP, Temperley WH, et al The dominant *Anopheles* vectors of human malaria in the Asia-Pacific region: occurrence data, distribution maps and bionomic precis. *Parasites and Vectors*.2011; 4(89): 1–46. 21612587 10.1186/1756-3305-4-89 PMC3127851

[10] World Health Organization. *World malaria report 2014* World Health Organization; 2014 December 2. Available from: http://apps.who.int/iris/bitstream/10665/144852/2/9789241564830_eng.pdf

[11] Rohani A, Lokman HS, Hassan AR, Chan ST, Ong YF, Abdullah AG, et al Bionomics of Anopheles balabacensis Baisas, the principal malaria vectors in Ranau, Sabah. *Tropical Biomedicine*.1999; 16(3): 1–8.

[12] World Health Organization. (2014*)* *WHO guidance for countries on combining indoor residual spraying and long-lasting insecticidal nets*. Geneva (Switzerland): WHO; 2014. 3p. WHO reference number WHO/HTM/GMP/MPAC/2014.2. Available from https://www.who.int/malaria/publications/atoz/who-guidance-combining-irs_llins-mar2014.pdf?ua=1

[13] World Health Organization. *Control of residual malaria parasite transmission*. WHO, Geneva, Switzerland 2014 9.

[14] Killeen GF. Characterizing, controlling and eliminating residual malaria transmission. *Malaria Journal*. 2014; 13(1): 330. 25149656 10.1186/1475-2875-13-330 PMC4159526

[15] Russell TL, Govella NJ, Azizi S, Drakeley CJ, Kachur SP, Killeen GF. Increased proportions of outdoor feeding among residual malaria vector populations following increased use of insecticide-treated nets in rural Tanzania. *Malaria Journal*. 2011; 10:80 10.1186/1475-2875-10-80 21477321 PMC3084176

[16] Durnez L, Moa S, Dennis L, Roelants P, Sochanta T, Coosemans M. Outdoor malaria transmission forested villages of Cambodia, *Malaria Journal*. 2013; 12(329), 1–14. 24044424 10.1186/1475-2875-12-329 PMC3848552

[17] Govella NJ, Ferguson H. Why use of interventions targeting outdoor biting mosquitoes will be necessary to achieve malaria elimination. *Frontiers in Physiology*. 2012; 3(199): 1–5. 22701435 10.3389/fphys.2012.00199 PMC3372949

[18] Russell TL, Beebe NW, Cooper RD, Lobo NF, Burkot TR. Successful malaria elimination strategies require interventions that target changing vector behaviours. *Malaria Journal*. 2013; 12(56): 1–5. 23388506 10.1186/1475-2875-12-56 PMC3570334

[19] Moiroux N, Damien GB, Egrot M, Djenontin A, Chandre F, Corbel V, et al Human exposure to early morning *Anopheles funestus* biting behavior and personal protection provided by long-lasting insecticidal nets. *PLoS ONE*. 2014; 9(8): 1–4. 10.1371/journal.pone.0104967 PMC4130624 25115830

[20] Rohani A, Zamree I, Wan Najdah WMA, Azahari AH, Matusop A, Zurainee MN, et al Impact of Indoor Residual-Sprayed Deltamethrin on Different Surfaces in a Malaria Endemic Area in Balai Ringin, Sarawak. *Advances in Entomology*. 2017;2: 151–160. 10.4236/ae.2014.23023

[21] World Health Organization. Report of the 16th WHOPES Working Group meeting. Geneva (Switzerland): WHO; 2013. 63p. Report No.: WHO/HTM/NTD/WHOPES/2013.6.

[22] World Health Organization. *Guidelines for testing mosquito adulticides for indoor residual spraying and treatment of mosquito nets*. Geneva (Switzerland): WHO; 2006. 70p. Report No.: WHO/CDS/NTD/WHOPES/GCDPP/2006.3.

[23] Reid JA. *Anopheline mosquitoes of Malaya and Borneo*. Malaysia: Institute for Medical Research Malaysia; 1968. 520p.

[24] Sallum MAM, Peyton EL, Harrison BA, Wilkerson RC. Revision of the Leucosphyrus group of *Anopheles* (Cellia) (Diptera, Culicidae). *Revista Brasileira de Entomologia*. 2005; 49:01–152.

[25] Vythilingam I, Lim YA, Venugopalan B, Ngui R, Leong CS, Wong ML, et al *Plasmodium knowlesi* malaria an emerging public health problem in Hulu Selangor, Selangor, Malaysia (2009–2013): epidemiologic and entomologic analysis. *Parasite Vector*. 2014;7(1):436. 10.1186/1756-3305-7-436 PMC4261908 25223878

[26] Fuehrer HP, Fally MA, Habler VE, Starzengruber P, Swoboda P, and Noedl H. Novel nested direct PCR technique for malaria diagnosis using filter paper samples. *Journal of Clinical Microbiology*. 2011; 49(4): 1628–1630. Available from: 10.1128/JCM.01792-10 21270224 PMC3122810

[27] Rubio JM, Post RJ, van Leeuwen WM, Henry MC, Lindergard G, Hommel M. Alternative polymerase chain reaction method to identify *Plasmodium* species in human blood samples: the semi-nested multiplex malaria PCR (SnM-PCR). *Transactions of The Royal Society of Tropical Medicine and Hygiene*. 2002; 96(1): 199–204. 12055839 10.1016/s0035-9203(02)90077-5

[28] Rohani A, Saadiyah I, Walgun A, Lee HL. Laboratory study on the effect of deltamethrin WG and WP formulations against *Anopheles maculatus* Theobald (Diptera:Culicidae) on rough and smooth surfaces of bamboo wall. *Tropical Bimedicine*.2007; 24(2): 77–82. 18209712

[29] Brooke B, Wood O, Koekemoer L, Mabuza A, Mbokasi F, Coetzee M. Small-scale field testing and evaluation of the efficacy and residual action of a new polymer-enhanced suspension concentrate deltamethrin formulation for malaria vector control in Mpumalanga province, South Africa. *Communicable Diseases Surveillance Bulletin* 2014;12(4): 108–114.

[30] Kijlstra J, Nentwig G, Rosenfeldt F, Sonneck R, Reid B, Gutsmann V. A polymer enhanced formulation to prolong the effectiveness of surface sprays. *Proceedings of the Eighth International Conference on Urban Pests*; 2014. Veszprem (Hungary): OOK-Press Kft; 2014 July. p. 383–388.

[31] Camilleri P. Alkaline hydrolysis of some pyrethroid insecticides. *Journal of Agricultural and Food Chemistry*. 1984; 32 (5):1122–1124.

[32] Demaute JP. A brief review of the environmental fate and metabolism of pyrethroids. *Pesticide Science*. 1989; 27(4):375–385.

[33] Santos RLC, Fayal ADS, Aguiar AEF, Vieira DBR, Povoa MM. Evaluation of the residual effect of pyrethroids on *Anopheles* in the Brazilian Amazon. *Rev Saude Publica*. 2007; 41(2): 276–283. 10.1590/s0034-89102007000200015 17384805

[34] Komalamisra N, Srisawat R, Apiwathanasorn C, Samsung Y, Kaisri P. Residual effect of 10% bifenthrin WP on mosquitoes and community acceptance, in eastern Thailand. *Southeast Asian Journal of Tropical Medicine and Public Health*. 2009 11 1;40(6):1221–1225. 20578456

[35] Mulambalah CS, Siamba DN, Ngeiywa MM, Vulule JM. Evaluation of lambda-cyhalothrin persistence on different indoor surfaces in a malaria epidemic-prone area in Kenya. *Research Journal of Biological Sciences*. 2010;5(3):258–63.

[36] Raghavendra K, Ghosh SK, Eapen A, Tiwari SN, Satyanarayan TS, Ravindran J, et al Field evaluation of lambda-cyhalothrin (ICON 10 CS) indoor residual spraying against *Anopheles culicifacies* in India. *Journal of Vector Borne Diseases*. 2011 3 1;48(1):18 21406733

[37] Yewhalaw D, Balkew M, Shililu J, Suleman S, Getachew A, Ashenbo G, et al Determination of the residual efficacy of carbamate and organophosphate insecticides used for indoor residual spraying for malaria control in Ethiopia. *Malaria Journal*. 2017 12;16(1):471 10.1186/s12936-017-2122-3 29162113 PMC5697437

[38] Dunford JC, Estep AS, Waits CM, Richardson AG, Hoel DF, Horn K, et al Evaluation of the long-term efficacy of K-Othrine® PolyZone on three surfaces against laboratory reared *Anopheles gambiae* in semi-field conditions. *Malaria Journal*. 2018 12;17(1):94 10.1186/s12936-018-2239-z 29471881 PMC5824574

[39] Durrheim DN, La Grange J, Hunt RH, Coetzee M, Govere J. Evaluation of the efficacy of deltamethrin using contact bioassays in a malaria vector control programme in Mpumalanga Province, South Africa. *African Entomology*. 2001 9 1;9(2):163–6.

[40] Reddy MR, Overgaard HJ, Abaga S, Reddy VP, Caccone A, Kiszewski AE, et al Outdoor host seeking behaviour of *Anopheles gambiae* mosquitoes following initiation of malaria vector control on Bioko Island, Equatorial Guinea. *Malaria Journal*. 2011 12;10(1):184. 21736750 10.1186/1475-2875-10-184 PMC3146901

[41] Siegert PY, Walker E, Miller JR. Differential behavioral responses of *Anopheles gambiae* (Diptera: Culicidae) modulate mortality caused by pyrethroid-treated bednets. *Journal of Economic Entomology*. 2009 12 1;102(6):2061–71. 10.1603/029.102.0607 20069832

[42] Badyaev AV. Stress-induced variation in evolution: from behavioural plasticity to genetic assimilation. *Proceedings of the Royal Society B*: *Biological Sciences*. 2005 5 7;272(1566):877–86. 10.1098/rspb.2004.3045 16024341 PMC1564094

[43] Jeffree SM, Ahmed K, Safian N, Hassan R, Mihat O, Lukman KA, et al Falciparum Malaria Outbreak in Sabah Linked to an Immigrant Rubber Tapper. *The American Journal of Tropical Medicine and Hygiene*. 2018 1 10;98(1):45–50. 10.4269/ajtmh.17-0081 29141714 PMC5928689

[44] Rohani A, Wan Najdah WMA, Mohd Hanif O, Aidil Azahary AR, Mohd Ariffin M, Zurainee MN, et al Characterization of the larval breeding sites of *Anopheles balabacensis* (baisas), in Kudat, Sabah, Malaysia. *Southeast Asian Journal of Tropical Medicine and Public Health*. 2018 7 1;49(4):566–79.

[45] Vythilingam I, NoorAzian YM, Huat TC, Jiram AI, Yusri YM, Azahari AH, et al *Plasmodium knowlesi* in humans, macaques and mosquitoes in peninsular Malaysia. *Parasites & Vectors*. 2008 12;1(1):26. 18710577 10.1186/1756-3305-1-26 PMC2531168

[46] Jiram AI, Vythilingam I, NoorAzian YM, Yusof YM, Azahari AH, Fong MY. Entomologic investigation of *Plasmodium knowlesi* vectors in Kuala Lipis, Pahang, Malaysia. *Malaria Journal*. 2012 12;11(1):213. 22727041 10.1186/1475-2875-11-213 PMC3476358

