# Supplementary figures and images for: Indoor and outdoor residual spraying of a novel formulation of deltamethrin K-Othrine® (Polyzone) for the control of simian malaria in Sabah, Malaysia
Source: PLoS One. 2020 May 15;15(5):e0230860. doi: 10.1371/journal.pone.0230860 (PMC7228059; doi:10.1371/journal.pone.0230860)

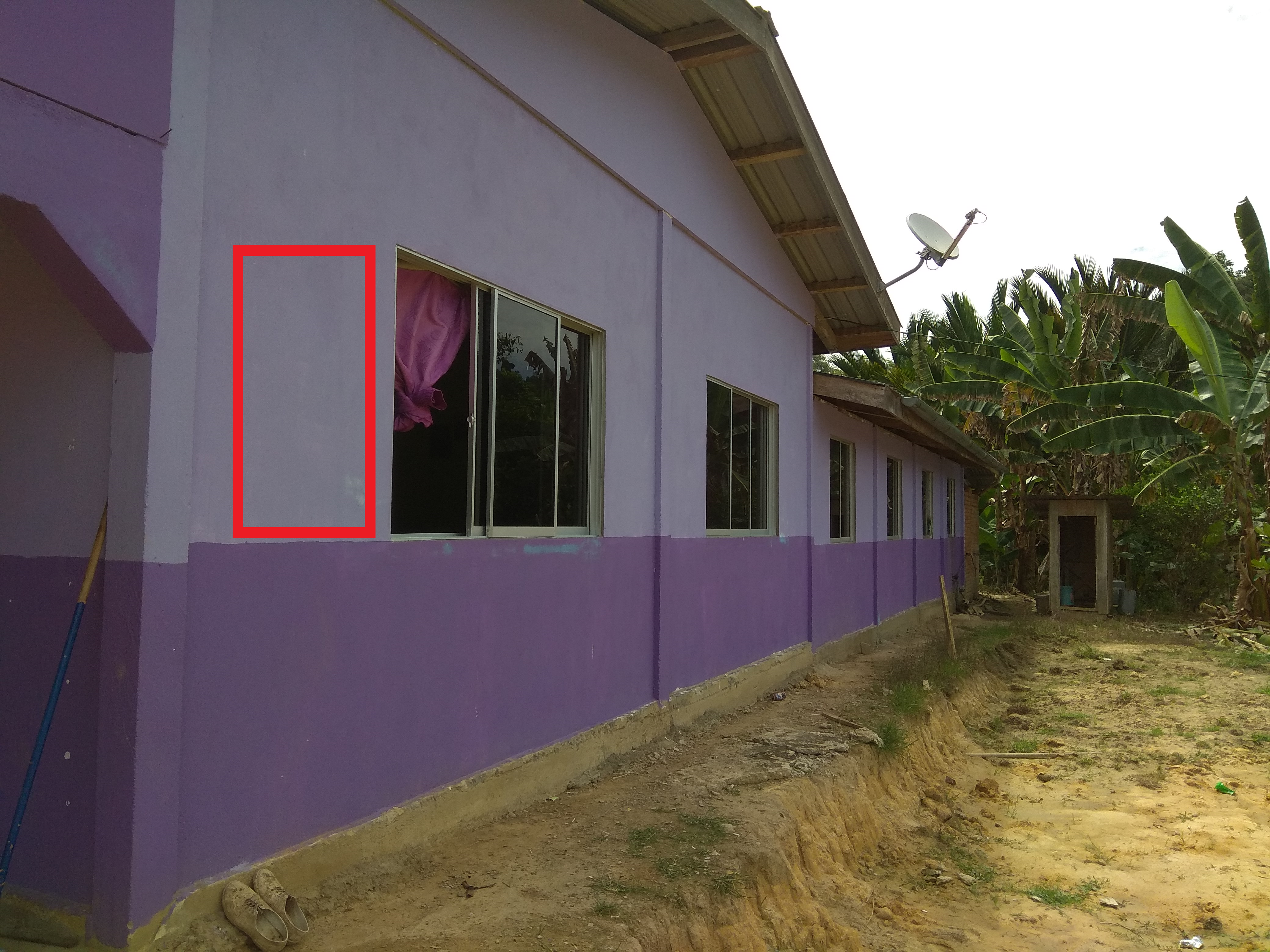

Supplement: S1 Fig — (TIFF) [file pone.0230860.s001.tiff]

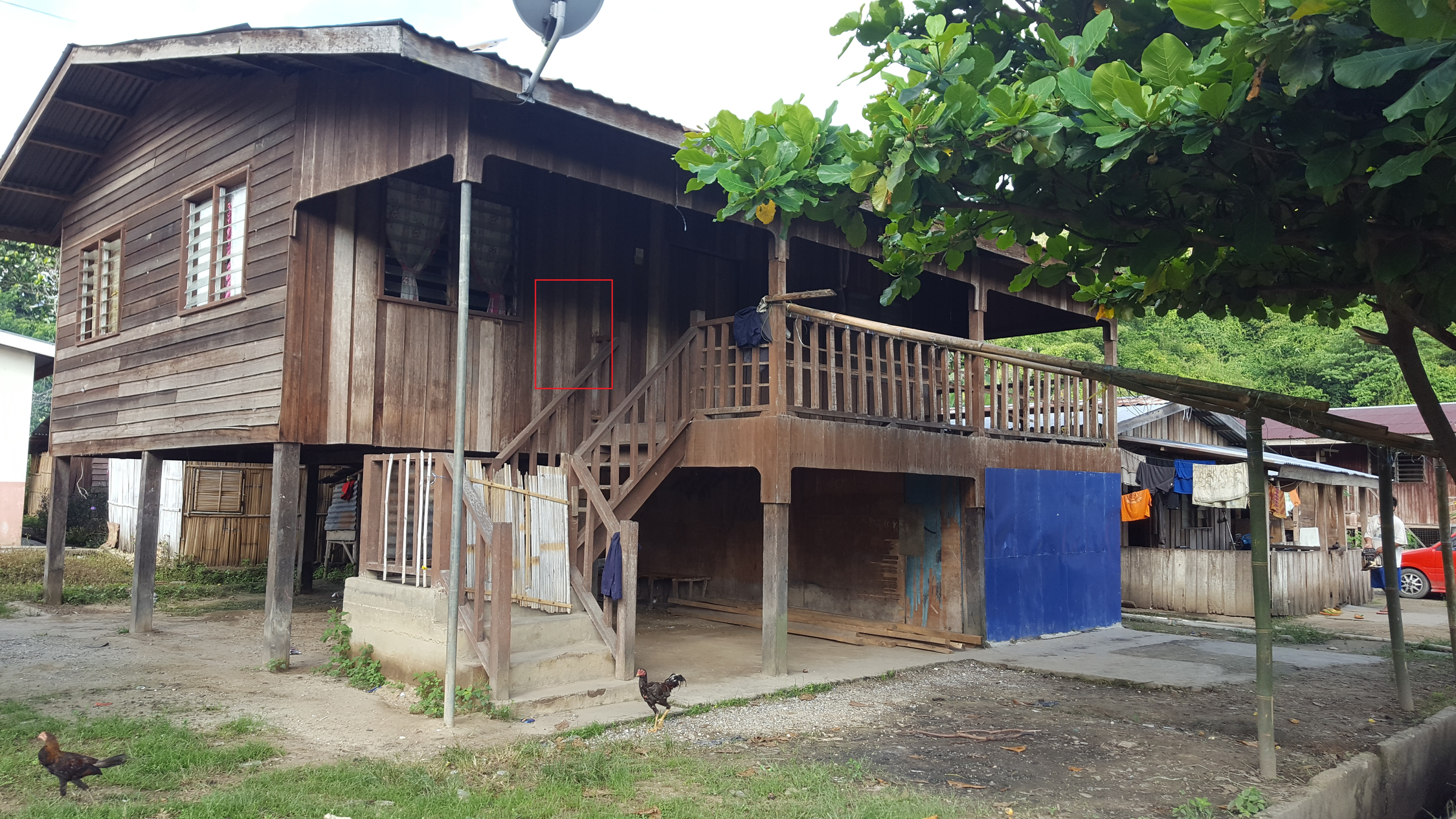

Supplement: S2 Fig — (TIF) [file pone.0230860.s002.tif]
