# Supplementary material for: Indoor and outdoor residual spraying of a novel formulation of deltamethrin K-Othrine® (Polyzone) for the control of simian malaria in Sabah, Malaysia
Source: PLoS One. 2020 May 15;15(5):e0230860. doi: 10.1371/journal.pone.0230860 (PMC7228059; doi:10.1371/journal.pone.0230860)
Supplement: S1 Raw Images — (PDF) [file pone.0230860.s003.pdf]

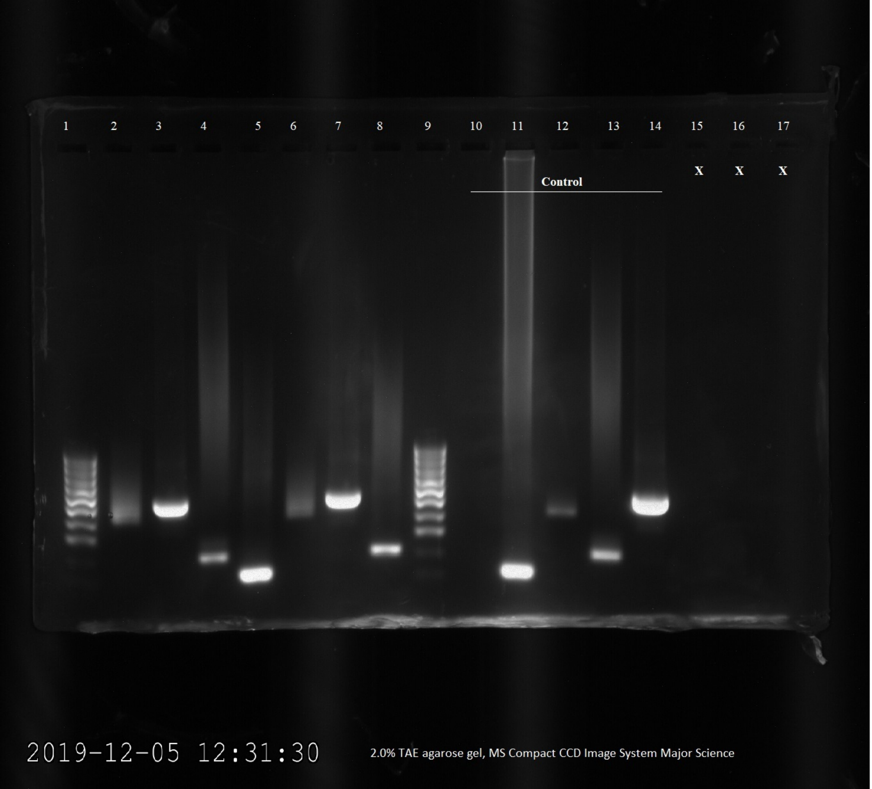

1. **100 Bp Ladder**
2. **004-pk**
3. **011-P sp.**
4. **016-pf**
5. **029-pv**
6. **037-pk**
7. **041-P sp.**
8. **050-pf**
9. **100 Bp Ladder**
10. **Negative Control**
11. ***P. vivax (121 Bp)***
12. ***P. knowlesi (450 Bp)***
13. ***P. falciparum (206 Bp)***
14. ***Plasmodium Sp. (600-700 Bp)***
15. ***X***
16. ***X***
17. ***X***

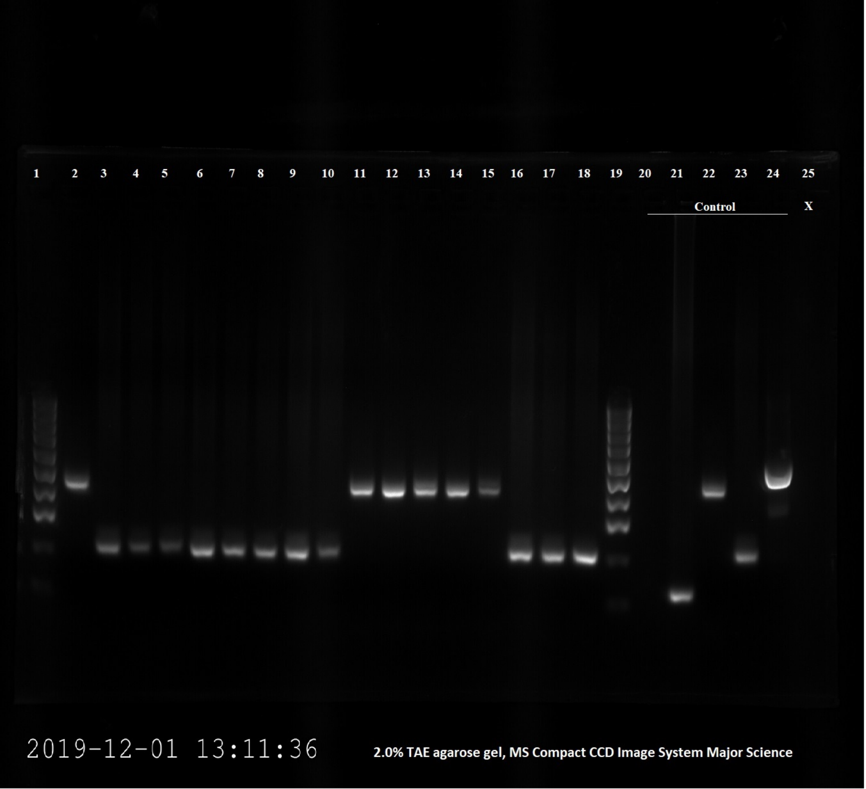

1. 100 Bp Ladder
2. 059-pk
3. 126-pf
4. 132-pf
5. 136-pf
6. 152-pf
7. 156-pf
8. 159-pf
9. 161-pf
10. 179-pf
11. 182-pk
12. 187-pk
13. 189-pk
14. 217-pk
15. 222-pk
16. 234-pf
17. 236-pf
18. 237-pf
19. 100 Bp Ladder
20. Negative Control
21. *P. vivax(121 Bp)*
22. *P. knowlesi (450 Bp)*
23. *P. falciparum (206 Bp)*
24. *Plasmodium Sp. (600-700 Bp)*
25. *X*

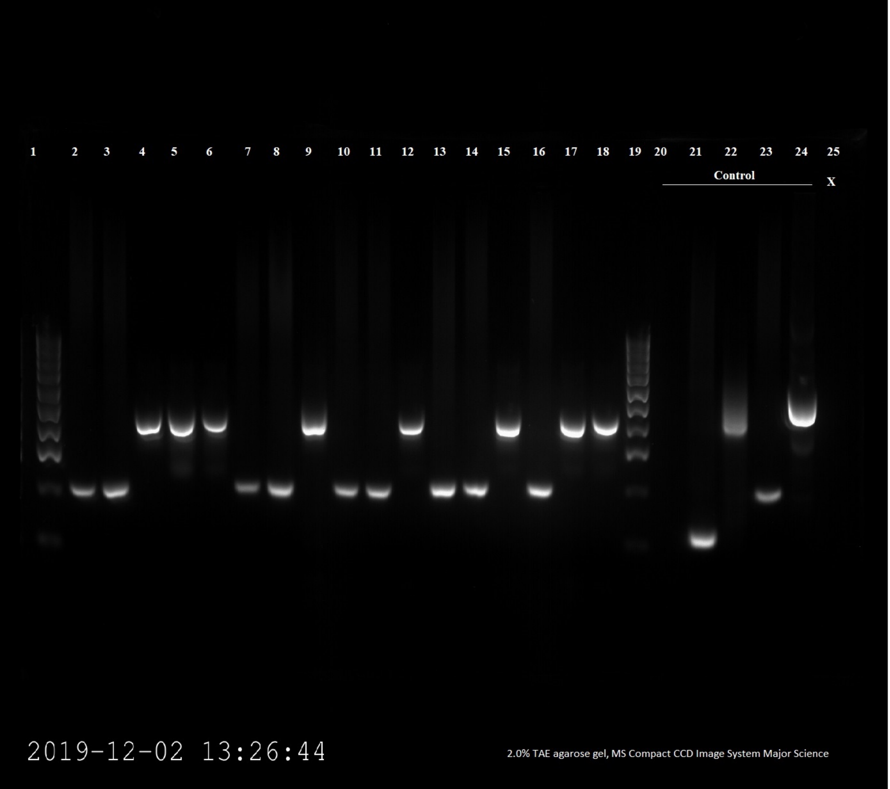

1. 100 Bp Ladder
2. 238 -pf
3. 240 -pf
4. 241 -pk
5. 242 -pk
6. 243 -pk
7. 244 -pf
8. 245 -pf
9. 246 -pk
10. 247 -pf
11. 248 -pf
12. 249 -pk
13. 250 -pf
14. 251 -pf
15. 252 -pk
16. 253 -pf
17. 268 -pk
18. 270 -pk
19. 100 Bp Ladder
20. Negative Control
21. *P. vivax(121 Bp)*
22. *P. knowlesi (450 Bp)*
23. *P. falciparum (206 Bp)*
24. *Plasmodium Sp. (600-700 Bp)*
25. *X*

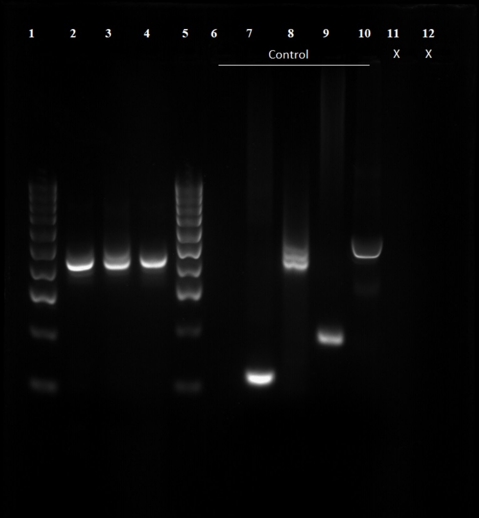

1. 100 Bp Ladder
2. 457-pk
3. 459-pk
4. 460-pk
5. 100 Bp Ladder
6. Negative Control
7. *P. vivax(121 Bp)*
8. *P. knowlesi (450 Bp)*
9. *P. falciparum (206 Bp)*
10. *Plasmodium Sp. (600-700 Bp)*
11. *X*
12. *X*

2019-12-02 17:08:13 2.0% TAE agarose gel, MS Compact CCD Image System Major Science
